# Georgia State Dental Society

**Published:** 1888-11

**Authors:** 


AMERICAN JOURNAL

—OF—

### DENTAL SCIENCE.

Vol. xxii. Third Series—NOVEMBER, 1888. No. 7.

ARTICLE I.

### GEORGIA STATE DENTAL SOCIETY.

BY MRS. M. W. J.

The Georgia State Dental Society convened in twentieth annual session, at Dalton, Georgia, August 22, 1888.

Officers: Drs. B. H. Patterson, President; S. A. White, 1st Vice-President; J. A. Chapple, 2nd Vice-President; W. L. Smith, Recording Secretary; L. D. Carpenter, Corresponding Secretary; H. A. Lowrance, Treasurer.

The President in his annual address confined himself to a review of the work accomplished by the society. To the work of societies is largely due the elevation of the profession to its present honorable position. Societies are organized and laws enacted in order to secure the greatest good to the greatest number. This was the object in view in the establishment of the Georgia State Dental Society and it has accomplished much good work; through the enactment of carefully considered dental laws the people have been freed from quacks and impositors, through whom they would otherwise suffer irreparable loss. The Executive Committee, also constituting the Board of Dental Examiners, has endeavored conscientiously to discharge its onerous duties in behalf of the people and the profession, though it has not always received the support and encouragement it deserves—many dentists and many of the people think it hard, unfair and unjust that a student who has passed through a dental college and received a diploma, should be obliged to pass a subsequent examination by a Board of Examiners before he is allowed to practice dentistry in the State of Georgia. But this only places the dental college on a par with other colleges. A graduate from Mucer University, Emery College, or any other literary institution is not allowed to engage in teaching until he has gone before the County School Commissioners and obtained a license.

The people should be educated to an appreciation of the services of a competent dentist, and to understand the value of the teeth. The State Society should educate, not only the dentist, but also the people.

Committees should be appointed not only to review addresses on dental education, and the theory and practice of dentistry for publication in the dental journals, but they should also have addresses to the public published in the papers of the day. This would not only benefit the people but it would also redound to the interests of the profession.

*Dr. Patterson*—While we rejoiced that the hand of death had spared all the active members of the society during the fifteen months which have elapsed since the last annual meeting it had yet removed a much esteemed friend, co-worker, and corresponding member, Dr. J R. Walker, of New Orleans: a dentist of the highest attainments, a gentleman of most amiable character ; those who knew him best loved him most. Dr. Patterson concluded with thanks for the honor conferred in placing him in the position of honor and trust he occupies, asking for the co-operation of all in making the meeting a successful one.

*Dr. Sid. Holland*—Spoke at some length on the great advances made in the science of *Chemistry*, and the value and importance of this branch of study to the dentist, though he could never hope to master it as it required the work of a lifetime: but he should master the elements of the science, especially in acquiring the new technology—when all use the same language each can understand the other.

*Dr. W. F. Tignor*—Spoke of the importance of a more thorough knowledge of *Therapeutics*, in enabling us to repair the ravages of disease and restore to usefulness the valuable organs under our charge. Too many remedial agents are used empirically—because they are recommended by some one else who has found them valuable. We should understand their therapeutic value, what they will accomplish, and in what way they act; we should be able to use our remedies scientifically and not blindly.

A paper from Prof. J. H. Coyle, Baltimore College of Dental Surgery, on the subject of *Dental Education* was read. Professor Coyle said that great improvements had been made in the line of college education; students were not turned out without at least a technical compliance with the rules of the Association of College Faculties. But the goal has not yet been reached; we have not yet attained a perfect system. Colleges are striving bravely to come up to the standard, and yet how many graduates are competent to take charge of a practice?

They cannot grasp and appropriate the pabulum offered them; they cannot execute with the hand that of which they have learned only the theory. College education is largely a course of memory-stuffing, looking only to the final examination and the coveted diploma. All the wisdom of Hippocrates will not make him a dentist unless he has a skilled hand; he must have the mechanical skill for a basis, and *time* is needed to cultivate and develop this.

To this end Prof. Coyle recommends that a two years' preliminary pupilage, under a competent preceptor, be made the basis of matriculation, hoping that the society would instruct her delegates to the National Board of Examiners to bring this matter up.

*Dr. H. S. Colding*—Said that he could not agree with Dr. Coyle. He had no preliminary pupilage but he remained through the summer sessions, and had all the work he could do in the infirmary, under competent instructors; far more so than if he had been in the office of a busy practitioner. A student who matriculates as late as possible in the session, and who leaves immediately after the examinations may need a precepter, but not otherwise.

*Dr. Holland*—Said we have but two ways of learning—what we find out onrselves, and what some one else tells us. The diploma only means "You are now fit to study without help." The old authors found out all they knew by practice or experiment. It makes no difference how a man learns a thing provided that he really knows it. According to the standards of to-day, it is better for a man to have a diploma, but he can be a successful dentist without it if he is determined and ambitious.

*Dr. S. A. White*—Said that if the ideas of the paper were carried out, it would exclude many of our best students from the colleges. A student is invariably placed first in the laboratory of his preceptor, and if the man has a large mechanical practice he will now get out of the laboratory, and will never see a tooth either extracted or filled. A first-class dentist with a large practice cannot personally instruct students; his practice will not permit it. Three years at college with full terms will give much better preparation.

*Dr.* S. *B. Barfield*—Said that he had been on the Examining Board of the State of Georgia for seven years, and he was highly gratified at the advances made in the colleges. Graduates now stand fully 33⅓ per cent. higher in their examinations than they did five years ago.

*Dr. Carpenter*—Said that the high standard of dental education to-day was due to the demands of State Societies through their Examining Boards.

*Dr. Cook*, Texas, Said that things were very much changed from his early days, when his preceptor paid $200 for the recipe for a "dental tincture" to kill the nerve of a tooth, and $300 for three weeks instruction from a traveling dentist from New Orleans.

*Dr. Main*, Dalton, a practitioner of medicine, being called upon, admitted the general indifference of medical men to diseases of the oral cavity; that dentists were striking the right line in tracing many lung and throat troubles to diseases of the mouth and teeth; he was satisfied that dentists could render great assistance to physicians.

Drs. Carpenter, Holland, Chapple, Thornton, Palmer, White, and others cited a large number of cases where physicians had diagnosed as cancer, osteo-sarcoma, mumps, etc., local trouble arising from uncerated roots, impacted wisdom teeth, etc., which after having been vainly treated for weeks and months by the physician, yielded readily after correct diagnosis by the dentist.

*Dr. D, D. Atkinson*, Brunswick, read a paper on The Dental Pulp, which he described as a most highly organized structure, the terminal point of arterial circulation, the dispenser of nutriment, the concentrator of all acute sensation, the seat of an intensity of pain that human endurance cannot stand, and for the relief of which dentistry is an absolute necessity.

In the discussion of this paper Dr. Crawford, Nashville, Tennessee, said that we do not fully understand the dental pulp. It is not, stated in the paper, the extremity of circulation nor a periphery of nerves; it is a ganglion, a bundle of nerves; it was not designed to be injured nor intended to transmit pain; it has no tactile power, no sense of heat or cold; it is capable only of transmitting a sense of injury to the brain. If exposed it is in violation of nature's laws. The dental nerve excites only reflex pain—it is nervous reflex action more than any thing else, like that of the iris to which it is wonderfully near of kin, but we do not understand its pathological conditions as thoroughly as does the oculist. Common traumatism, or septic conditions may be relieved and the nerve restored to perfect health. We can diagnose a pulp-stone, but we cannot cure it. The oculist can do more than that with the iris. It is a fortunate thing for Humanity that the life of the nerve is not of vital importance for the preservation of the tooth, but that pulpless teeth when properly filled may do good service for many years.

*Dr. W. H. Weaver*—From notes gave the history of a number of interesting cases of replantation.

*Dr. H. S. Colding*—Had no faith in replantation. If a tooth was too frail to fill in the mouth, it was too frail to extract successfully. He would in all cases crown with Logan or other crown. He had been very successful in the treatment of dead teeth, treating with iodoform and wood creosote paste, changing it every other day, sealed with Gilbert's patent filling material.

*Dr. F. L. Adair*—Described a very interesting case in which he asked the advice of older practitioners. The patient is a boy of eleven years of age, who from the age of one month up to nine years old had been an invalid, with convulsions, etc. Did not erupt any teeth until after he was three years old. He now has all of the permanent teeth due at his age, but they are very extensively decayed with nerve exposures, and so soft as to be indented with the finger nail. Two of the first permanent molars have been extracted, the other two are decayed below the margin of the gums. The two superior canines are only partially grown, but are already badly decayed. The bicuspids alone might possibly be saved. Shall they all be extracted at that early age? They cannot be filled, they cannot be crowned, they are all badly diseased.

*Dr. W. H. Morgan*, Nashville, Tennessee, said it was evidently an extreme case of faulty development. The teeth had evidently never been fully organized. All those that cannot be saved should be removed. It is not good practice to preserve devitalized molars at that age. The promotion of the general health and development of the body might improve the teeth somewhat, but all the teeth of soft structure, with pulp exposed, should be removed, or grave results might ensue. Tooth structure at that age contains so much animal matter that putrefaction ensues, with abscess and septic poisoning. The bicuspids and molars may be lost early yet leave no permanent gap in the arch. If taken out at the age of twelve or fourteen the space will fill up and at twenty-five the loss will not be felt.

*Dr. Tignor*—Asked what he would do with little baby teeth when early decayed?

*Dr. Morgan*—Take them out.

*Dr. Adair*—Asked how if the decay before the roots have begun to absorb?

*Dr. Morgan*—Take them out.

*Dr. S. A. White*—Said that with all due respect to Dr. Morgan he could not sit still and hear such indiscriminate extraction recommended. He thought we should make every possible effort to preserve the deciduous teeth, until the natural time for their loss.

*Dr. Morgan*—Said that after the death of the pulp there would be no physiological absorption of the root and nature would never get rid of them. A devitalized deciduous tooth cannot remain in the mouth a month without producing disease and danger to the permanent teeth, either destroying the germ or causing imperfections of the enamel, through the pathological condition of the surrounding membranes.

*Dr. White*—Did not believe the permanent teeth were universally affected by decayed deciduous teeth. We often see devitalized temporary teeth, with the crowns all broken off, followed by perfect permanent teeth.

*Dr. Morgan*—Said that in every case of disease we should remove the cause of disease.

*Dr. J. Y. Crawford*, Nashville, Tenn., said there was no question of greater importance than this, and it grieved him to hear this indiscriminate extraction [illegible]ciduous teeth recommended. There was no matter of greater importance than the retention of the deciduous teeth, from the canines to the molars. It is true there is no absorption after devitalization. The extraordinary physiological functions are always the most susceptible. An organ which has but one work to perform—a specific work—is the most susceptible. A large cavity, even without nerve exposure, may interfere with and retard absorption. But we have no means to supplement that physiological function. He said, I believe in my heart if the dental profession was not capable of managing and controlling such cases, I would resign and quit. We supplement that function how? by keeping them isolated and non-antagonizing; they will then exfoliate, permanent dentition may be retarded, but not interfered with. Permanent dentition is often too precocious, and there is no harm done in retarding it. If the germs of the permanent teeth are in contact with putrefaction the result will be bad, but keep them free from that, and the tooth will be perfectly organized. It is not even necessary that it should ever see the light for perfect development, as is seen in teeth developed in transverse position. If the tooth is diseased I treat it; if it is devitalized I treat it; there is no condition we cannot treat; establish drainage till restoration is brought about; treat then as we do the permanent teeth; keep them isolated and non-antagonizing. I have retained them thus for three years, and had the new teeth from below in good condition.

*Dr. Catching*—Wished to enter a protest against the views of Dr. Morgan, as not being the views of the Georgia State Dental Society, and to endorse the position of Dr. Crawford.

*Dr. Morgan*—Said that he did not intend to say to extract all deciduous teeth, when they were doing no harm, and giving no trouble; they may be tolerated if there is no decided disease.

*Dr. Catching*—Said he would also disagree with Dr.

Morgan in regard to systemic treatment and supplying bone material. He would give abundance of sweet milk and lime, and food especially selected for bone material.

*Dr. Morgan*—Asked what food could possibly be selected that did not have ample bone constituents? Even the poorest flour contains 2¼ per cent. which, at the rate of half pound per day would give 2¼ pounds in a year, nearly the weight of the entire osseous system of a child of that weight, Professor Huxley putting the weight of the dried human skeleton at 6 to 8 pounds.

*Dr. Catching*—Said the system would probably not appropriate the whole amount furnished.

*Dr. Morgan*—Did not think it would assimilate inorganic matter until it had passed through animal life.

*Dr. Catching*—Asked him about iron in concentrated form as administered by physicians?

*Dr. Morgan*—Said that it was not assimilated by the human system but remained mineral iron still.

*Dr. Catching*—Thought it made little difference what shape it was in so that it accomplished the work for which it was administered. He did not believe in the chemical-laboratory-of-the-animal system to convert it into pabulum.

The subject was passed and Dr. H. H. Johnson, of Hawkinsville, read a paper on "Devitalized Teeth."

He said that the difficulties surrounding the treatment of devitalized teeth, tax the skill, the ability, and the utmost resources of the dental practitioner; it is a subject that often looms up as a stumbling block in our path. In his treatment, when he devitalizes a tooth, he waits a week after making the application, giving time for the dead tissue to slough, which it will do at the apex if time enough is given. Then adjusting the rubber dam he cuts freely through the crown surface, to gain access. A fine Donaldson nerve cleaner is then pushed cautiously into the canals and given half-dozen turns when the nerve will be caught by the barbs and can be brought out entire in the majority of cases. After cleaning as thoroughly as possible with this, he enlarges the orifice with a large sized Gates Glidden drill in the engine, going about one-third of the way down. He does not claim that it will follow a curved canal, but it will come as near to it as anything can, and if cautiously handled there is very little danger of breaking. It should be frequently withdrawn and cleaned, to prevent clogging, or it may get fastened in the canal and twisted off. With a smaller size he enlarges nearly to the apex, if straight. If it turns hard he " suspicions " a curve and goes no further. After the upper third has been enlarged the remaining debris is readily cleansed away. He gives several reasons for enlarging the canal: 1st, the canal cannot be filled if too small for the passage of a broach. 2nd, it cuts out all dentine of which the tubuli may be infiltrated, with putrescent water, and 3rd, it makes the canal round and easier to fill. After cleaning he dries with warm air, and pumps full of eugenol or eugenic acid, using this exclusively.

If there is no fistulous opening he makes one, and rarely treats an abscess; nature will attend to that if the cause is removed.

If the tooth has been devitalized for some time, and decomposition has set in with incipient pericementitis, he is more cautious. He cleans thoroughly at the first sitting, and fills the roots with cotton saturated with eugenol, stopping the crown cavity to exclude the moisture, giving instructions to call at once if any soreness follows. If it is all right at the end of a week or ten days he dries with warm air and fills; never allows water or saliva to enter the canal after the first cleaning unless the apical foramen is unusually large and moisture has entered there. Microbes and spores do not thrive in a dry locality. In the latter case the cotton in the root is frequently changed, with fresh medicine to prevent mephitic gases. In this class of teeth the pumping motion must be avoided or moisture will be drawn in. If there is an opening for drainage immediate filling is permissible. He fills the canal with oxy-chloride, which is disinfectant, and has a drying effect on the dentine.

He uses eugenol exclusively in the treatment of roots because it has no escharotic qualities, does not coagulate albumen, creates no irritation if pumped through, has an odor of cloves which is not objectionable, it penetrates the tubuli and checks putrefactive changes ; it has all the good qualities of carbolic acid and none of the bad.

In the discussion of this paper Dr. Catching said he did not find it so extremely easy to remove the root contents entire, as Dr. Johnson seemed to. He had tried the Gates Glidden drill once, as he thought very cautiously, but the first thing he knew he went out through the side of the root into the gum and brought blood ; he prefers to leave the canals as he finds them. He had been filling root canals with chlor-percha, as he supposed successfully, until on one occasion recently, wishing to remove a portion for a permanent filling he pulled out the whole thing, from both roots and the pulp chamber, and each root lacked one-half of being filled to the apex.

*Dr. H. S. Colding*—Applies arsenic for twenty-four hours, followed by wood creosote for twenty-four hours ; this mummifies the pulp and it comes out entire. He had used two Gates Glidden drills and broke them both off in the tooth, having to rust them out with salt. On one occasion his filling went through the apex and caused trouble, but the use of aconite, iodine and chloroform, and Darby's plaster cured it.

*Dr. Morgan.*—Characterized Dr. Johnson's paper as a most admirable one, which has rarely been equalled for clearness and conciseness. He also commended his method and treatment. He considers the Gates Glidden drill a snare and a fraud—they will pop off and play the old nick. So also the Morey drill to a certain extent. He recommends caution in the use of warm air, too much drying will impair the bond of union between dentine and enamel ; the dentine will draw away from the arched cups of the enamel and the latter will break away peeling off like the bark from a tree.

All antiseptics coagulate, some more than others. It is to this property that bichloride of mercury owes its efficiency.

*Dr. S. A. White*—Thinks that as a rule we treat too much. No one agent will answer in every case. He uses bichloride of mercury and listerine most frequently. Agrees with Dr. Morgan as to the Gates Glidden drill; broke one once off and had to extract the tooth; no matter how cautiously used they will pop off.

*Dr. Johnson*—Said he had used the same two exclusively for a long time. He takes a size larger than the canal, turns cautiously and cleans frequently.

*Dr. W. S. Smith*—Broke one off and had to extract the tooth. He used salt and iodine and worked at it for weeks, then concluded to risk filling with the steel in, but it soon gave trouble and he had to extract the tooth. He uses a Donaldson nerve broach and cleans as thoroughly as possible, using peroxide of hydrogen followed by bichloride of mercury, then iodoform and eucalyptus, stopping it up for a day or two. If it gives no trouble he fills in a few days. He fills the roots with oxy chloride mixed very thin and pumped up with bibulous paper on a broach.

*Dr. Tignor*—Said he had used oxy-chloride for the last time unless he could find something to keep it from going through. It was apt to stir up a little volcano.

*Dr. Holland*—Pumps it up with a soft gold wire and leaves the wire in to close the foramen. He flattens the wire and bevels it at the end. He never reams out the canal.

*Dr. R. W. Thornton*—Never drills out the canal, it only complicates the matter, you are too liable to find crooks and curves, and to go through. He pumps carbolic acid through the fistulous opening, using floss silk and a soft broach. If the canal is too small to admit a broach it does not need filling.

*Dr. White*—Thinks Dr. Tignor's trouble due to air forced through the foramen. He uses a hickory point to close the foramen, and considers long staple cotton better than floss silk.

Dr. Crawford recommended sterilized orangewood for root filling. He considered the paper of Dr. Johnson one of the best he had ever heard and was much pleased with the discussion. The original object in saving pulpless teeth was merely to prevent a gap. Now that they are so largely utilized as supports for bridgework their importance is greatly enhanced. There are three classes of cases, three conditions of dead teeth—a simple devitalized tooth, without complication; a blind abscess, or incomplete fistula, and a complete fistula. In the first case every teaching of minor surgery inculcates immediate filling. There are three conditions which may give trouble; 1st, an imperfectly filled canal, leaving a reservoir for the accumulation of offensive matter; 2nd, the passage of offensive matter beyond the apex—either a broken instrument or filling material; and 3rd, the pumping through of decomposing septic matters. The potency of virus is best illustrated in the process of vaccination, when a particle invisible to the naked eye, introduced through a tiny scratch from the point of a cambric needle, may permeate the entire system. The material used for filling is immaterial so that you get it where you want it. The first thing is to close the apical foramen; get it down to a fine point and yet not pass through. If it is corked up at that end it may be left open at the other end and washed and treated as much as you please, so that the walls of the canal are not softened. In the treatment of abscess we don't appreciate the importance of refrigeration; the reduction of temperature, either by ice or ether spray.

Dr. Morgan wished to say a few words on the importance of opening the canals (especially in patients who have reached the meridian of life) when there will often be found deposits of osteo-dentine in the efforts of nature to protect the pulp. This must be cut through to reach the canal. The end of the root should be reached through the fistulous opening and smoothed off. It will often be found roughened by dissolved cementum.

On the subject of Prosthetic Dentistry Dr. Campbell spoke at length in favor of vulcanite rubber.

Dr. E. F. Adair read a paper on Artificial Dentures.

Dr. Crawford spoke with great animation on the subject, deploring the downward tendency of mechanical dentistry through the introduction of cheap rubber work and the consequent reckless sacrifice of teeth which might have been made to do good service for years. This unnecessary extraction is the cause of shortening of life in the United States, a potent factor preying upon our influence and making ours a profession of paupers. He said: I thank God for the genius that suggested bridgework, for an Allen who gave us continuous gum work.

Dr. Holland said he would not wipe out vulcanite altogether. It was like the hickory shirt and jeans pants of the working man. All cannot wear fine linen and broadcloth, neither can all have gold or continuous gum work. There are those who must have work that can be made for a low price, and to them rubber plates are a boon.

Dr. Thornton said that the introduction has degraded dentistry in that it had pulled down prices so that it was degrading to a man of skill and scientific ability to work at the prices that now rule in prosthetic dentistry.

Dr. White said that in spite of all that could be said against it, rubber had come to stay, the best thing we could do with it was to persuade all our patients to have it gold-lined. It is more healthful to the mouth, and commands a better price.

Dr. Holland spoke of the injurious effects of bridgework upon the adjoining teeth.

Dr. Crawford thought movable plates much more harmful to the adjoining teeth than bridgework.

Dr. Morgan said that if impressions were left in a bowl of water for ten minutes (after being varnished) the cast would have a smooth hard surface such as could not be produced by any other method.

By request, Dr. J. Y. Crawford spoke of the influence of erupting wisdom teeth in producing diseases of the respiratory tract.

He said that the results of close observation in this direction were very striking. Tonsilitis, inflammation of the mucous membrane lining the throat, even phthisis pulmonalis could all be traced to difficult eruption of the wisdom teeth. He said that he was an advocate for the preservation of all the teeth—non-breaking of the dental arch, except in the case of hereditary irregularity, but if any teeth must be sacrificed they should be the wisdom teeth of the upper jaw, making the lower ones non-antagonizing.

In many common local throat affections, the primary lesion is of a traumatic nature, from the closing of an upper cusp on the lower jaw causing a pinching and hardening of that structure. From that indurated tract a line of inflammation passes back to the tonsils, glandular masses whose secretions are designed to lubricate the respiratory tract. From inflammation they fail to perform that function, and the throat is chafed and ulcerates, and permanent disability, a chronic affection ensues.

Pathological tissue cannot be absorbed. It is changed from normal to hyperplastic and is never gotten rid of.

We speak of first and second dentition, but there is no such thing; it is one complete operation from embryonic to adult life, and the same conditions though in less degree may ensue from the eruption of the sixth year and the twelfth year molars, a tendency to hypertrophy. Barring out hereditary tendency to phthisis pulmonalis, a large per cent. of lung troubles are due to the difficult eruption of the wisdom teeth; the primary lesion is around the lower tooth, or where the upper tooth strikes the lower jaw, spreading thence to the tonsils, throat, and air passages. This is a subject to which our young men would do well to devote special attention. This little thing remedied may enable a patient to avoid premature decline. There is nothing like oral and dental surgery; it leads the world. If carried fully up to all it is capable of, we may yet become the teachers of the world in pathology.

Dr. L. D. Carpenter thinks that fully ninety per cent. of all cases of catarrh and other nasal affections, affections of the eye and ear, of the throat and lungs, and even gout, palsy and rheumatism are due to diseases of the mouth and teeth.

Dr. Morgan said that every departure from a physiological condition of an organ or structure impairs more or less the efficiency of the function it is designed to perform.

Aberrations from the normal condition of the mucous membrane of the posterior portions of the oral cavity are much more frequently due to the eruption of the first and second molars than has been supposed. Diseased secretions pass over the delicate membranes of the larynx and pharynx which are readily affected by these abnormal fluids. Every departure from health in the oral cavity affects these organs. We have healthy pus first, but through continued inflammation it is changed and becomes offensive, excoriating the throat and causing serious trouble. This should be closely watched and cared for. The medical world is at last waking up to the importance of diseases of the mouth and teeth. A distinguished medical man recently said that "If the race becomes edentulous as is prophesied, it will wipe out all the business of the dentist and half of that of the physician."

Dr. Gordon, a physician of Dalton, was called upon. He said that he was glad to express his high appreciation of dental surgery, but he was somewhat startled at learning the immense domain claimed by them. He recognized that a broken tooth might cause an affection resembling cancer which would be cured by extracting the tooth. A neuralgic eye or ear-ache may be cured by extracting a diseased tooth, but he was not prepared to yield to them the splendid domain of otology and ophthalmology. It is true that we cannot have perfect health without perfect digestion, nor perfect digestion without perfect mastication, nor perfect mastication without perfect teeth, so that in the last analysis the health of the people depends on the skill of the dental profession, and it looks as if they would occupy the whole field, following the alimentary tract to its other extremity and treating even hæmorrhoids.

Dr. Gordon spoke of the indebtedness of the medical world to the dental profession for that great discovery—anæsthesia, the greatest boon ever conferred upon the human race, and yet, strange to say, by far the greater per cent. of accidents from its use occur in the practice of dentistry—so many cases of "death in the dental chair" are reported.

Dr. Morgan agreed that it was much safer in capital operations than in minor surgery. Excitement and suffering severe pain being an antidote to all anæsthetics. All anæsthetics occasionally produce death. There have been six cases of death from cocaine reported in the last few days. He thinks anæsthetics not safe for dental operations.

Thc hour for the election of officers having arrived the election was held with the following result: Dr. S. A. White, President; Dr. W. Y. Tignor, First Vice-President; Dr. J. A. Thornton, Second Vice-President; Dr. L. D. Carpenter, Cor. Secretary; Dr. H. H. Johnson, Rec. Secretary; Dr. H. A. Lowrance, Treasurer.

Tybee was selected as the next place of meeting, the regular time being the second Tuesday in May, 1889.—*Reported for Dental Register.*

